# Systemic tumor regression with synergy therapy: radiotherapy and CAR-T

**DOI:** 10.1038/s41420-024-02245-3

**Published:** 2024-11-22

**Authors:** Xingyu Ma, Wei Zhang, Miao Zeng, Teeranut Asavasupreechar, Synat Kang, Yisheng Li, Li Yu

**Affiliations:** 1 Department of Hematology and Oncology, Shenzhen University General Hospital, International Cancer Center, Hematology Institution of Shenzhen University, Shenzhen University Health Science Center, Shenzhen Clinical Research Center for hematologic disease, Shenzhen University, Shenzhen, China; 2 Biomedical Laboratory, Shenzhen University-Haoshi Cell Therapy Institute, Shenzhen, China; 3 R&D Department, Shenzhen Haoshi Biotechnology Co., Ltd, Shenzhen, China

**Keywords:** Cancer immunotherapy, Immunization

## Abstract

Pancreatic ductal adenocarcinoma (PDAC) is one of the most poorly prognostic digestive tract malignancies. CLDN18.2 CAR-T therapy has recently shown promising clinical effects in PDAC. Radiotherapy, a traditional treatment, can induce systemic immune activation and abscopal effects. However, the synergistic effect and mechanism of their combination in PDAC treatment remain poorly understood. In this study, we developed a CLDN18.2-specific CAR-T and applied it to unilateral and bilateral mouse tumor models. Our results demonstrated that this synergy therapy not only improved tumor-killing effects in unilateral tumor-bearing mice but also induced regression in both local and distant tumors in bilateral tumor models. Mechanistically, early radiation-induced apoptosis promoted the proliferation of CD8 + T cells, while increased chemokine CCL2 levels from localized and distant tumor sites facilitated CAR-T and endogenous T cell infiltration, leading to systemic tumor suppression. This study proposes a promising approach for treating metastatic pancreatic cancer by combining radiotherapy and CAR-T therapy, elucidating the mechanism of CAR-T cell-enhanced radiotherapy effects ex vivo, and highlighting a novel strategy for combating metastatic pancreatic cancer.

## Introduction

Pancreatic ductal adenocarcinoma (PDAC), abbreviated as pancreatic cancer, remains one of the most lethal malignancies, with a dismal 5-year survival rate of less than 10%. This grim statistic ranks PDAC as the sixth leading cause of cancer-related mortality worldwide [1]. Despite the potential curative benefits of surgical intervention, this option is available only to a small subset of patients diagnosed with resectable tumors. Furthermore, surgery alone is insufficient for addressing advanced and metastatic tumors, which account for over 80% of PDAC diagnoses at the time of detection [2]. For patients with advanced pancreatic cancer, systemic chemotherapy remains first-line therapy, but median survival is still less than one year [3]. Thus, developing new strategies to treat advanced pancreatic cancer holds paramount clinical significance and poses a globally recognized challenge.

Chimeric Antigen Receptor T-cell (CAR-T) therapy represents a groundbreaking approach in cellular immunotherapy, wherein T cells are genetically engineered to express receptors that specifically recognize antigens on cancer cells. This targeted strategy has shown remarkable success in treating certain hematologic malignancies, such as B-cell lymphomas and acute lymphoblastic leukemia [4]. However, translating this success to solid tumors, including PDAC, remains challenging despite its promise. A recent Phase I trial of CLDN18.2 CAR-T therapy in gastrointestinal cancers showed an impressive objective response rate (ORR) of 45.3%. In contrast, the pancreatic cancer cohort had an ORR of only 20% [5].

One of the primary obstacles in CAR-T therapy for solid tumors is the hostile and dense tumor microenvironment (TME). The TME of PDAC is particularly desmoplastic, characterized by a dense extracellular matrix, fibroblasts, immunosuppressive cells, and a hypoxic environment [6]. These factors collectively impede the infiltration, persistence, and efficacy of CAR-T cells within the tumor mass. Furthermore, pancreatic tumors often exhibit heterogeneity in antigen expression, leading to challenges in consistently targeting cancer cells [7, 8].

Radiotherapy (RT) emerges as a promising adjunct to CAR-T therapy, offering a multifaceted approach to overcoming the formidable barriers posed by the TME in solid tumors like pancreatic cancer. Additionally, RT has demonstrated substantial benefits for patients with metastatic cancer through the intriguing phenomenon known as the abscopal effect. Ionizing radiation induces DNA damage, oxidative stress, and cell death [9], which collectively stimulate the immune system. Antigen-presenting cells (APCs) recognize tumor-associated antigens (TAAs) released by irradiated cells [10, 11]. In response, primary dendritic cells (DCs) undergo maturation, migrate to the lymph nodes, and present TAAs to CD8 + T lymphocytes (CTLs), thereby initiating an antitumor immune response against both primary and metastatic tumors [12, 13]. Thus, RT may modulate the TME by reducing physical and immunological barriers that hinder CAR-T cell infiltration.

In this study, we demonstrate that combining radiotherapy with CAR-T therapy yields superior antitumor effects compared to monotherapy. One key reason for this enhanced efficacy is the increased proportion of CD8 + T cells within the CAR-T cell population. Additionally, through bilateral tumor loading experiments, we provide evidence that the combination of radiotherapy and CAR-T therapy exerts significant inhibitory effects on both local and distant tumors. We also verify that radiotherapy enhances the expression of genes associated with T cell migration, thus promoting the distal killing effect of CAR-T cells. This work suggests a promising approach for treating metastatic pancreatic cancer by combining radiotherapy and CAR-T therapy and elucidates the mechanisms by which CAR-T cells enhance the abscopal effects of radiotherapy.

## Results

### CLDN18.2-specific CAR T cells preparation

**Fig. 1:**
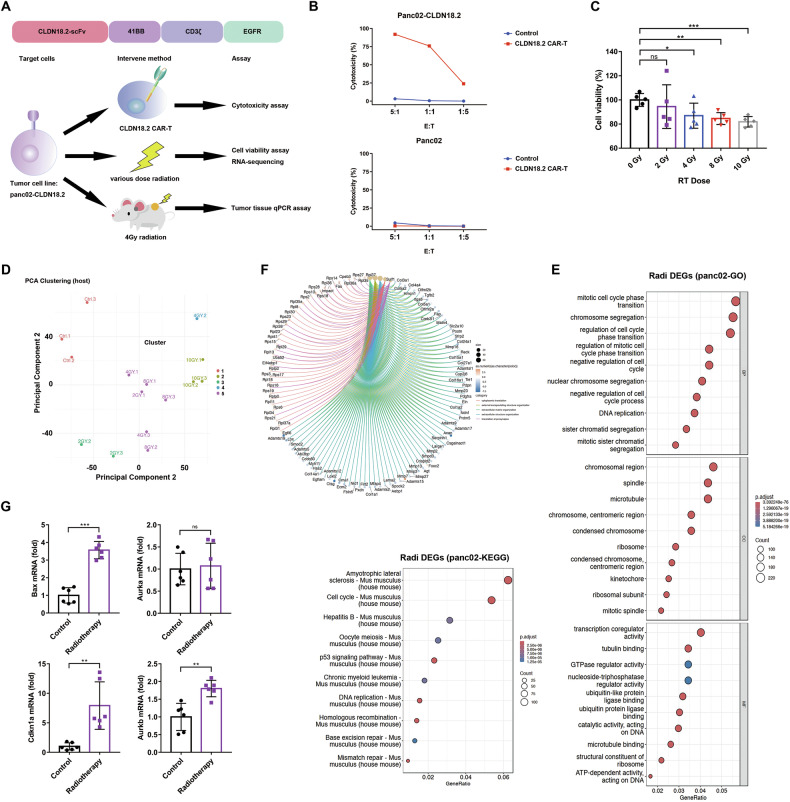
Induction of pancreatic cancer cells killing by CLDN18.2 CAR-T and RT. **A** Schematic of the vectors encoding CLDN18.2 CAR and experimental plan of events. **B** overexpressed CLDN18.2 panc02 or wild panc02 cells were mixed with CLDN18.2 CAR or control T cells at various effector: target ratios for 18 h followed by quantification of target cell killing. **C** Tumor cell viability 48 hr after exposure to various doses of radiation. CLDN18.2 panc02 cells were exposured to various doses of radiation (0 Gy, 2 Gy, 4 Gy, 8 Gy, 10 Gy), the cell viability was detected by CCK-8 assay after 48 h. **D** Principal component analysis of gene expression of target cells 6 h after various doses of RT. **E** Enrichment analysis for gene sets in GO and KEGG in the differentially expressed genes characteristic for the RT groups. Colors in the scatter diagram represent the level of significance of the enrichment (−log 10 of the adjusted p values). **F** Clustering and gene expression network chart (red, high; blue, low) with names of strongest genes discriminating the non-RT group. **G** BAX, CDKN1A, AURKA and AURKB mRNA expression in the tumor samples after exposure RT. Data in (**C**, **G**) presented as the mean ± SD, *n* = 5–6, statistically significant differences were calculated by two-tailed Student’s *t* test. *p < 0.05, **p < 0.01, ***p < 0.001.

Claudin18 (CLDN18) is an integral membrane protein present in the tight junctions of epithelial and endothelial cells, existing in two isoforms: CLDN18.1 and CLDN18.2. Claudin18.2 expressions in is highly expressed pancreatic cancers, and occasionally in bile duct, ovarian, colorectal, and pulmonary tumors under pathological conditions [14]. To specifically target PDAC, we constructed a CLDN18.2-specific single-chain variable fragment (scFv) named F2H. Firstly, to verify its specificity, we created CHO cell lines overexpressing either CLDN18.1 or CLDN18.2. The F2H CLDN18.2 CAR-T cells exhibited cytotoxicity only against the CLDN18.2-expressing cells and not the CLDN18.1-expressing cells (Fig. S1C and S1D). Due to the low expression levels of CLDN18.2 in commonly used pancreatic cell lines, we overexpressed CLDN18.2 in the pancreatic cancer cell line Panc02 (Fig. S1A and S1B). As shown in Fig. 1B, F2H CLDN18.2-specific CAR T cells demonstrated effective cytotoxicity against pancreatic cancer tumor lines expressing CLDN18.2. These data indicate that F2H CLDN18.2 CAR T cells possess intrinsic target-dependent cytotoxic activity.

### Anti-tumor activity of radiotherapy

To verify the cytotoxicity of RT, we first irradiated tumor cells with various doses and performed cell viability assays and RNA-sequencing analyses (Fig. 1A). The cell viability assay confirmed that 4 Gy is the minimum dose required to induce significant tumor cell death (Fig. 1C). Principal component analysis demonstrated clear separation between control and irradiated cells based on apoptosis-related genes (Fig. 1D). Compared with the control, pathway analysis revealed that radiation enriched differentially expressed genes (DEGs) in cell cycle and DNA replication pathways (Fig. 1E). The circular plot in Fig. 1F further illustrates the differential gene expression and pathway enrichment analysis in response to RT. These data verified that CLDN18.2-expressing Panc02 cells are sensitive to radiotherapy, and cell proliferation signals are altered upon RT.

Further analysis revealed that the effect of RT on tumor cells was not primarily dose dependent. Key genes related to apoptosis and cell cycle regulation, including Bax, Cdkn1a, Aurkb, and Aurka, exhibited saturation in expression levels around 10 Gy. This suggests that increasing the radiation dose beyond this point does not significantly enhance the expression of these critical genes (Fig. S2A–S2C). Therefore, we selected the lowest effective dose of 4 Gy for subsequent experiments. Next, we established a mouse model of heterogeneous solid tumors by subcutaneously injecting CLDN18.2-expressing Panc02 cells. One week later, the mice were treated with 4 Gy RT, and tumor samples were collected after 7 days (Fig. 1A). Among those highly upregulated genes affected by RT in cell cycle and DNA replication pathways, Bax, Cdkn1a, and Aurkb were validated in qRT-PCR of these tumor samples (Fig. 1G). These results demonstrate that 4 Gy radiation can exhibit anti-tumor activity both in vitro *and* in vivo.

### Local irradiation combined with CAR-T cells enhances tumor suppression by upregulating CAR-T cells

**Fig. 2:**
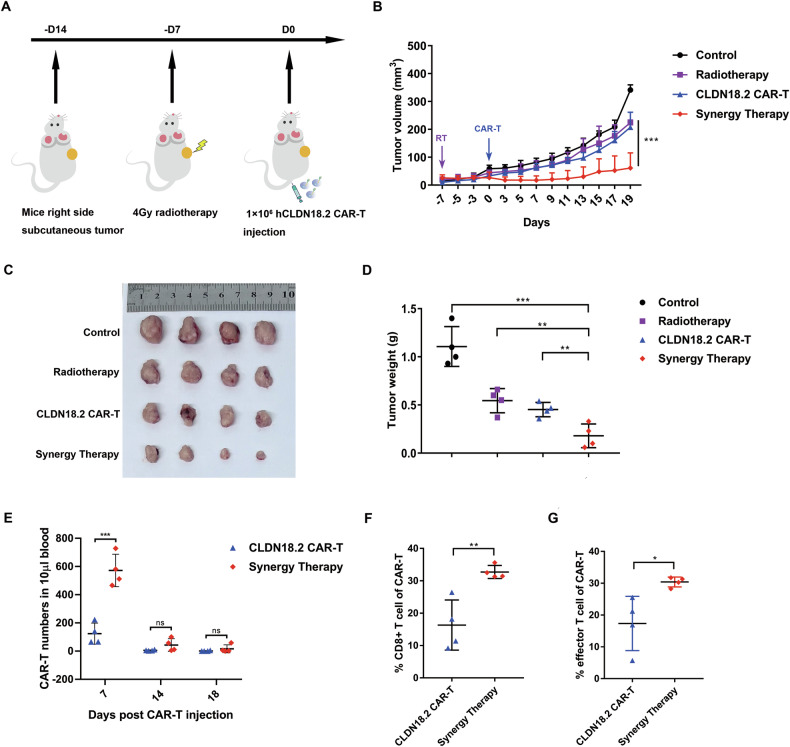
Synergy therapy delays tumor growth through increasing CD8 + CAR-T In Vivo. **A** NCG mice were injected CLDN18.2 panc02 subcutaneously on the right side. Seven days later, the mice were separated to 4 groups (*n* = 4), two groups were randomly selected to treat with 4 Gy local radiotherapy, on the 14th day, and the mice were intravenous injected with control T cells or CLDN18.2 CAR-T cells. **B** The tumor volumes were recorded every two days and analyses using the formula $\pi \div6\times H\times W\times L$, multiple comparisons were performed with two-way ANOVA. **C** Tumors of mice were assessed at the time of necropsy with representative macroscopic images, and (**D**) the tumor weight was measured. **E** Murine peripheral blood was drawn at 7, 14, 18 days after indicated CAR-T cell infusion to determine the CAR-T cell counts. Data indicate the mean number of T cells per 10 μl of blood as measured by flow cytometry. Evaluation of CD8 + CAR-T (**F**) and effector CAR-T cell percentage (**G**) 7 days post CAR-T injection. Data in (**D**–**G**) were calculated by two-tailed Student’s *t* test. presented as the mean ± SD, *n* = 4. *p < 0.05, **p < 0.01, ***p < 0.001.

We previously demonstrated that CLDN18.2-expressing Panc02 cells are sensitive to both radiation and CLDN18.2-specific CAR-T therapy independently. To evaluate the combined effect, we established a mouse pancreatic cancer model treated with both CAR-T cells and radiotherapy (RT). As shown in Fig. 2A, CLDN18.2-expressing Panc02 cells were injected subcutaneously on the right flank of the mice. Seven days later, the mice received 4 Gy of local irradiation, followed by CAR-T cell infusion another seven days later. Our results indicated that while 4 Gy local irradiation or CLDN18.2 CAR-T treatment alone slowed tumor regrowth, the synergy therapy of RT and CAR-T significantly delayed tumor regrowth (Fig. 2B). Representative images demonstrated that the synergy therapy significantly reduced tumor size (Fig. 2C) and also resulted in a notable decrease in tumor weight (Fig. 2D).

To detect the proliferation of CAR-T in mice, we measured the amount of CAR-T in peripheral blood at different time points after CAR-T infusion. Increased CAR-T cells were observed in the tumors treated with the synergy therapy 7 days post-CAR-T infusion compared to tumors treated with CAR-T alone (Fig. 2E), although all number of CAR-T cells decreased over time. Additionally, CD8 + T cells and effector T cells, which are significant indicators of CAR-T efficacy, were also analyzed. The results showed that RT increased the percentage of CD8+ positive CAR-T cells (CD3 + CD8 + EGFR + ) and effector CAR-T cells (CD3 + EGFR + CD45RA + CCR7-) in peripheral blood 7 days after CAR-T injection, highlighting the enhanced anti-tumor activity of the synergy therapy. (Figs. 2F, G and S3A). Thus, combining radiation with CAR-T therapy led to an increase in the number of CAR-T cells and a higher proportion of CD8+ positive T cells, resulting in enhanced suppression of tumor cell proliferation and survival.

### Synergy therapy achieved superior tumor suppression in both local and abscopal lesions

**Fig. 3:**
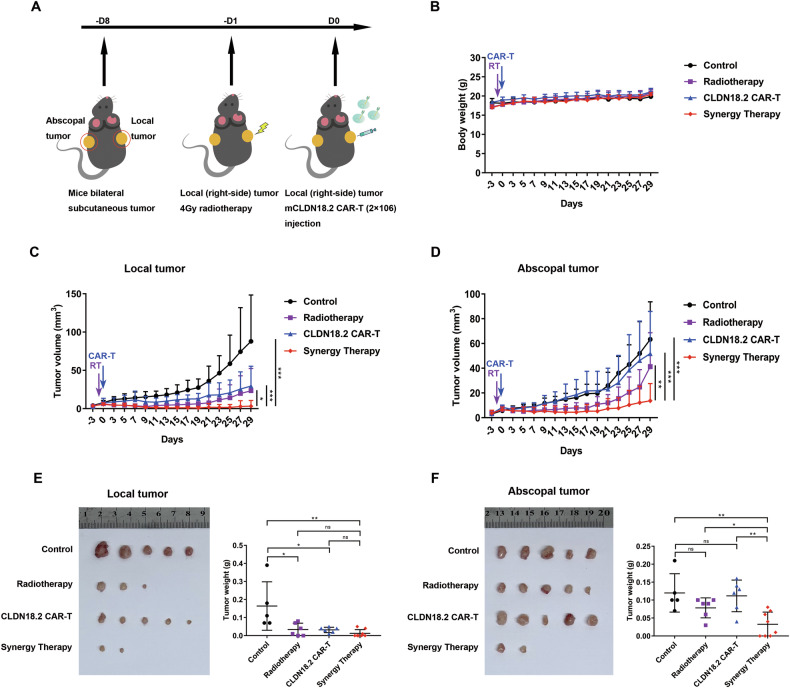
RT and CAR-T synergy therapy inhibits both local and abscopal tumor growth. **A** Combine treatment schema in bilateral tumor model. C57BL/6 mice were implanted with CLDN18.2 panc02 on the left (2 × 10^6^, on day 0) and right (2 × 10^6^, on day 0) lower sides of the abdomen, and received treatment on day 7 and 8. The body weight (**B**) were recorded. Tumor growth curves represent the average volume of (**C**) local (treated, right-side) or (**D**) abscopal (untreated, left-side) tumors in each group. Data are represented as mean ± SD, the control group *n* = 5, radiotherapy group *n* = 6, CLDN18.2 CAR-T group *n* = 6 and synergy group *n* = 8. Two-way ANOVA was used for statistical analysis. ns, not significant representing, *p < 0.05, **p < 0.01, ***p < 0.001. The representative macroscopic images (left) of local tumors (**E**) and abscopal tumors (**F**) were shown, and the tumor weight (right) was measured separately.

To extend our previous work in immunodeficient mice, we utilized C57BL/6 mice bearing tumors derived from CLDN 18.2-expressing Panc02 cells to create a syngeneic model. This allowed us to monitor the systemic antitumor benefits, particularly the influence on the host immune system, of our strategy combining radiotherapy and CLDN18.2 CAR-T cells (Fig. 3A). In this model, tumor cells were implanted on both the right and left sides of the mice to evaluate both local and abscopal effects. To avoid the host immune system attacking the CAR-T cells after injection, we isolated T cells from the spleens of C57BL/6 mice and generated CLDN18.2 CAR-T cells through retroviral transduction. These syngeneic CLDN18.2 CAR-T cells also demonstrated specific and effective cytotoxicity against CLDN18.2-expressing Panc02 cells (Fig. S4A).

In the experimental setup (Fig. 3A), CLDN18.2-expressing Panc02 cells were implanted on both sides of the mice. Seven days later, the right-side tumors received RT treatment, followed by CAR-T cell injection one day later. Our results showed that for local (right-side) tumors, a complete regression rate of 33% (2/6) was achieved with radiotherapy monotherapy compared to the control group. Tumor suppressive effects were further enhanced when radiotherapy was combined with CAR-T therapy, achieving a complete regression rate of 63% (Fig. 3C, E). While CAR-T therapy alone caused significant regression of tumors at the locally treated site, it was not effective on abscopal (untreated) lesions. The radiotherapy seems have slight abscopal effects without significant difference. Notably, our synergy strategy significantly enhanced tumor suppression on abscopal lesions (Fig. 3D, F). Additionally, body weights of the mice were recorded during treatment, and all groups showed similar patterns, indicating the considerable safety of the synergy strategy (Fig. 3B).

### CAR-T infiltration both in local and abscopal tumor induced by RT and CAR-T synergy therapy

**Fig. 4:**
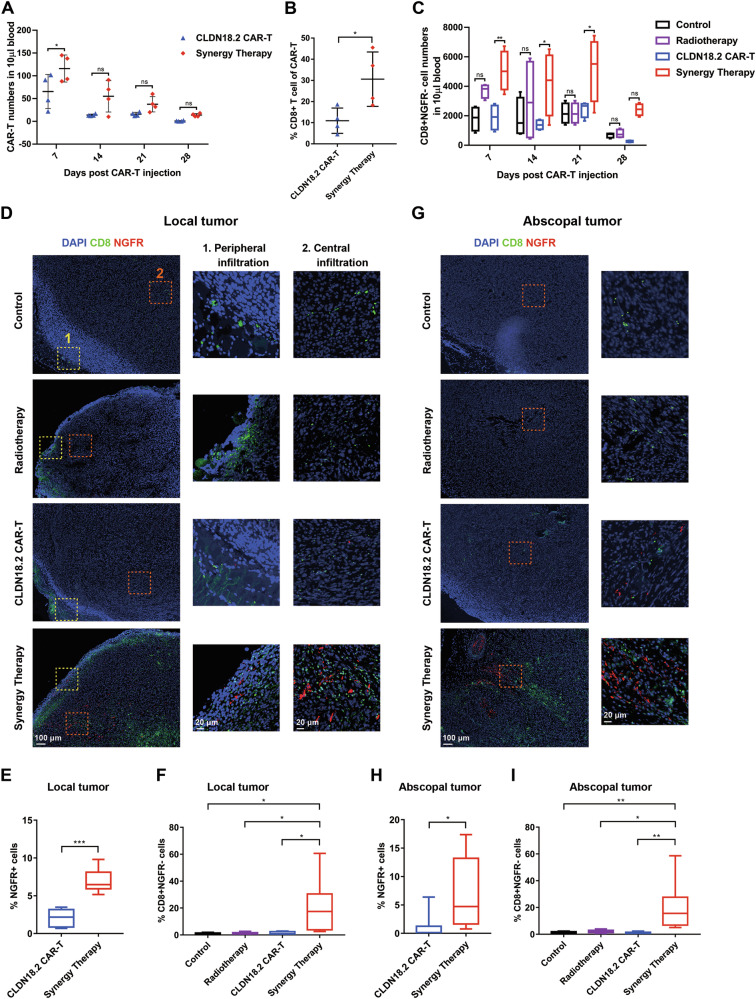
CAR-T infiltration induced by synergy therapy. Numbers of CAR-T cell (**A**) and CD8 + NGFR- cell (**C**) in murine peripheral blood was drawn at indicated after CAR-T cell infusion. Data indicate the mean number of T cells per 10 μl of blood as measured by flow cytometry (*n* = 4). **B** Proportion of CD8 + CAR-T in murine peripheral blood 7 days post CAR-T injection. Multispectral immunostaining of local tumor (**D**) and abscopal tumor (**G**) for DAPI (blue), CD8 (green), NGFR (red). Quantification of NGFR+ cells (**E** (local tumor), **H** (abscopal tumor)) and CD8 + NGFR- cells (**F** (local tumor), **I** (abscopal tumor)) central infiltration by Image J. Statistically significant differences were calculated by two-tailed Student’s *t* test. Data in (**B**, **C**, **E**, **F**, **H** and **I**) presented as the mean ± standard deviation, *n* = 4. *p < 0.05, **p < 0.01, ***p < 0.001.

Similar to the findings in immunodeficient mice, murine peripheral blood collected from the syngeneic model exhibited higher levels of CAR-T cells in the synergy therapy group compared to the CAR-T therapy alone group. The number of CAR-T cells increased moderately when combined with radiotherapy, with a significant upregulation in the proportion of CD8+ cells (30.6%) in the RT + CAR-T group at 7 days post-CAR-T infusion (Figs. 4A, B, S5A). As inferred from studies on the “abscopal effect” of RT [15], the systemic antitumor immune response should be activated after treatment. Therefore, we further examined the levels of host endogenous T cells in each group. There was almost no difference in the number of the total host endogenous T lymphocytes (CD3 + NGFR-) in peripheral blood between each group with the proportion of CD8+ significantly upregulated in synergy groups (Fig. S5B, S5C) in the early stage (day7). For the total times of the endogenous T cells (CD3 + CD8 + NGFR-), it was strikingly increased in the synergy therapy group, and was remained elevated for several weeks (Fig. 4C). This result suggesting that the introduction of radiotherapy significantly enhanced the inhibitory efficacy towards distant lesions.

To understand the spatial distribution of tumor-infiltrating T cells, which is essential for tumor eradication, we applied immunofluorescence staining to tumor sections at the end time point (28 days). We assessed the infiltration of endogenous CD8 + T cells (CD8 + NGFR-) and CAR-T cells (NGFR + ) on both local and abscopal tumor slides. Though a slight increase in endogenous CD8 + T cells was detected in the blood of the RT group mice at early stages (Fig. 4C). Then, immunohistochemistry of CD8 was performed with early tumor tissues, and it was found that the expression of CD8 was increased in both the radiotherapy group and the synergy therapy group compared with the control group, indicating that radiotherapy may promote the infiltration of CD8 cells in the early stage (Fig. S5D and S5E). There were no apparent differences among the control, CAR-T, and RT groups for endogenous CD8 + T cells at the endpoint (Fig. 4C). However, a slightly increased number of endogenous CD8 + T cells in peripheral tissue was observed at local side (Fig. 4D), suggesting a limited role of radiotherapy in long-term tumor eradication. In contrast, in the synergy group, the number of endogenous CD8 + T cells increased dramatically in both local and abscopal tumors (Fig. 4F, I). Similar results were observed for CAR-T cells in the local site (Fig. 4D–F), indicating that radiotherapy enhances endogenous T cell infiltration, which is further boosted by CAR-T therapy. Additionally, radiotherapy significantly increased the number of CAR-T cells infiltrating abscopal tumors (Fig. 4G–I), highlighting the substantial synergistic effect of combining CAR-T and radiotherapy.

### Synergy therapy boosting of systemic tumor suppression relies on increased cell migration signals

**Fig. 5:**
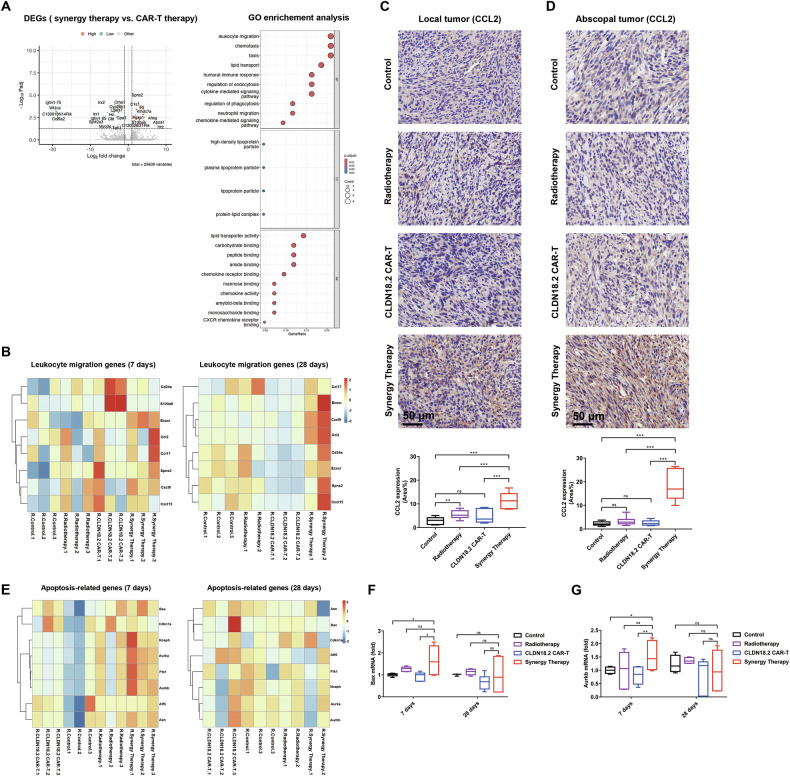
RT and CAR-T synergy therapy up-regulated the expression of CXCL9 and CCL2 in local tumors. **A** Volcano plots (left) of gene expression CAR-T monotherapy (left) and combine (right) in local tumor 28 days post CAR-T infusion (*n* = 3). All genes were showed using –log10(FDR) and log2(fold change) values and the red dots (up) and the green dots (down) represent genes with > 2fold change in expression and FDR < 0.01. Enrichment analysis (right) for gene sets in the differentially expressed genes characteristic for the RT + CAR-T groups. Colors and size in the scatter diagram represent the level of significance of the enrichment (−log 10 of the adjusted *p* values). **B** expression heat maps of right-side (local, treated) tumors in control group, RT group, CAR-T group, and CAR-T + RT group 28 days and 7 days post CAR-T treatment. GSEA software (Broad Institute) were used to calculate nominal *P* values and FDR q values. Immunohistochemistry of local tumor (**C**) and abscopal tumor (**D**) for CCL2. Quantification of CCL2 expression (area%) by Image J. **E** expression heat maps of right-side (local, treated) tumors in control group RT group, CAR-T group, and CAR-T + RT group 28 days and 7 days post CAR-T treatment. GSEA software (Broad Institute) were used to calculate nominal *P* values and FDR q values. qRT-PCR quantification of cell death related genes bax (**F**) and aurkb (**G**) in control group, RT group, CAR-T group, and CAR-T + RT group, data were normalized to housekeeping gene GAPDH. Statistically significant differences were calculated by two-tailed Student’s *t* test. Data in (**C**, **D**, **F** and **G**) presented as the mean ± SD, *n* = 4–8. *p < 0.05, **p < 0.01, ***p < 0.001.

To explore the tumor-associated immune changes following the synergy therapy, we performed NGS RNA sequencing. In the local tumor site, 47 genes were significantly upregulated and 67 genes were downregulated in the synergy therapy group compared to CAR-T group at 28 days post-CAR-T injection, as shown in Fig. 5A. GO enrichment analysis demonstrated a significant enrichment of leukocyte migration-related genes and chemotaxis genes. We compared these related gene expression profile at 7 days and 28 days across the control, RT, CAR-T, and synergy groups (Fig. 5B). These significant gene expression differences were detected at 28 days, particularly in genes such as chemokine CCL2 and CXCL9, indicating that these migration-related genes play a crucial role at later stages, while no significant trends were observed in the synergy group at 7 days.

To further validate these molecular signatures, we performed immunohistochemical (IHC) staining on tumors at the endpoint (28 days). IHC staining for CCL2 at both local and abscopal sites at 28 days revealed a marked increase in CCL2 expression at both sites (Fig. 5C, D), while no significant difference was observed for CXCL9 (Fig. S6A–D). Quantitative analysis showed that the proportion of CCL2 expression increased from 4.72 ± 1.02% in the CAR-T group to 11.48 ± 1.91% in the synergy group in the local tumor. The difference also detected in abscopal tumor from 2.54 ± 0.38% in the CAR-T group to 18.46 ± 2.30% in the synergy group (Fig. 5C (left) and 5D (right), respectively). This suggests a positive correlation between T cell increase and CCL2 expression.

Additionally, we observed an increase in the expression of cell death-related genes related to RT in tumor tissues during the early phase of synergy therapy (7 days) (Fig. 1G). However, no significant differences were found in tumor tissues at 28 days, indicating that irradiation-induced cell death occurs predominantly in the early stages (Fig. 5E–G). Overall, these data demonstrate that early tumor suppression is primarily due to synergy therapy-induced apoptosis, while late distal tumor regression is attributed to CCL2-mediated T cell migration.

## Discussion

In this study, we confirmed that combining radiotherapy with CAR-T cells enhances systemic tumor regression. Moreover, our data provide insight into the synergistic mechanism of RT and CAR-T-cell therapy. Initially, tumor damage from radiotherapy induces the proliferation of CAR-T cells and endogenous CD8 + T cells. Subsequently, in the later stages, increased expression of the chemokine CCL2 in both localized and distant tumors leads to the infiltration of myeloid cells and T cells. This process further amplifies the immune response, ultimately resulting in systemic tumor regression.

Radiotherapy causes damage to tumor cells, leading to the release of damage-associated molecular patterns (DAMPs). These DAMPs can activate local immune cells and induce an inflammatory response and the formation of a “hot tumor”. The abscopal effect is a phenomenon of the systemic inflammatory response caused by radiotherapy and was first reported in 1953 [16]. However, its biological mechanism is not fully understood, and the lack of adequate understanding of its mechanisms has been a major limitation of its combination. CD8 + T cells play a significant role in recognizing and attacking both primary tumors and metastatic diseases [17, 18]. On this basis, we hypothesized that the combination of modified CAR-T cells with RT would increase this effect. Although several preclinical and clinical studies have shown that synergistic therapy has a stronger effect than does monotherapy [19–23], few studies have elucidated the relationship between CAR-T-cell therapy and abscopal effects. Our study suggested that RT induced early damage to tumor cells, while the addition of CAR-T cells further increased the inflammatory response and promoted the generation of a systemic immune response.

Unlike B cells, T cells usually need to come into direct contact with antigen-carrying cells to perform their effector functions. Therefore, controlling the migration of CD8 + T cells to solid tumors and reaching cancer cells is the key to improving patient survival and the immunotherapy response. RT combined with CAR-T cells can stimulate the production of chemokines by immune cells (such as macrophages and dendritic cells), endothelial cells, and even the tumor cells themselves, including C-C motif chemokine ligand 2 (CCL2) and C-X-C motif ligand 9 (CXCL9). CCL2, also known as monocyte chemoattractant protein (MCP)-1, primarily interacts with CCR2+ monocytes, memory T cells, and dendritic cells to mediate their activation and migration [24]. CXCL9 is known for its role in recruiting T cells, particularly Th1 cells and cytotoxic T lymphocytes (CTLs) [25], to the site of inflammation. In addition, CCL2 and CXCL9 expression is regulated by the NF-κB signaling pathway [26, 27]. Thus, synergistic therapy may induce inflammatory activation, resulting in increased CCL2 secretion and the recruitment of APCs (such as DCs). CXCL9 released by DCs increased the infiltration of CD8 + T cells at the tumor site. The increased expression of these chemokines helps in the recruitment of immune cells to irradiated tissue, which can enhance the antitumor immune response.

Modulating the TME is a key area of focus in cancer treatment. In particular, the TME of pancreatic cancer is extremely complex. The TME refers to the cellular environment in which a tumor exists and consists of various noncancerous cells (such as immune cells, fibroblasts, and endothelial cells), extracellular matrix components, and signaling molecules [28]. By altering the expression of chemokines such as CXCL9 and CCL2, radiotherapy can modulate the TME, increasing the likelihood of an effective immune response against the tumor. Furthermore, the coexpression of the CCL2 receptor CCR2b [29] or CXCL9 [30] in CAR-T cells can enhance the infiltration and tumor killing ability of CAR-T cells, which can lead to improved tumor control and potentially enhance the efficacy of synergistic therapies involving radiotherapy and immunotherapy. This chemokine axis guides the interaction between T cells and DCs within the tumor, highlighting a new approach to restoring sensitivity to immune checkpoint inhibitors [31]. Additionally, the combination of radiation therapy and the addition of a blocker to the immune checkpoint with CAR-T cells has the potential for systemic tumor elimination.

In summary, this study aimed to elucidate how local radiotherapy restructures the immune microenvironment and enhances CAR-T-cell-mediated cytotoxicity at local and distant tumor sites. Our research has substantial translational potential considering that local radiotherapy combined with CAR-T cells may provide new theoretical foundations for clinically effective treatment strategies for advanced metastatic solid tumors.

## Materials and methods

### Reagents

The monoclonal antibodies (mAbs) used for flow cytometry included the following: APC/Cyanine7 anti-human CD3, PerCP/Cyanine5.5 anti-human CD4, PE/Cyanine7 anti-human CD8, Brilliant Violet 605™ anti-human CD45RA, FITC anti-human CD197 (CCR7), APC anti-human EGFR, APC/Fire™ 750 anti-mouse CD3, Brilliant Violet 510™ anti-mouse CD8, APC anti-mouse CD4, and PE/Cyanine7 anti-human CD271 (NGFR) were purchased from Biolegend (San Diego, CA, USA). Anti-p75 NGF receptor antibody (ab52987) and anti-CXCL9 antibody (ab202961) were purchased from Abcam (Cambridge, MA, UK). The CD8α (D4W2Z) XP® Rabbit mAb was purchased from Cell Signaling Technology (Danvers, MA, USA). The anti-CCL2/MCP-1 antibody (A7277) was purchased from ABclonal (Wuhan, China). The agents used in this study included the following: recombinant human interleukin-7/15/21 (PeproTech, Rocky Hill, NJ, USA), the EasySep™ Human T-Cell Isolation Kit (Stemcell, Vancouver BC, Canada), the ImmunoCult™ Human CD3/CD28/CD2 T-Cell Activator (Stemcell, Vancouver BC, Canada), the MojoSort™ Mouse CD3 T-Cell Isolation Kit (Biolegend, San Diego, CA, USA), the Dynabeads Mouse T-Activator CD3/CD28 (ThermoFisher, Waltham, MA, USA), the GeneJuice® Transfection Reagent (Merck, Rahway, NJ, USA), GlutaMAX™ (Gibco, GrandIsland, NY, USA), β-mercaptoethanol (Gibco, GrandIsland, NY, USA), polybrene (Sigma, St.Louis, MO, USA), puromycin (Sigma, St.Louis, MO, USA), the recombinant fibronectin fragment (Retronectin; Takara Shuzo, Japan), and the CytoTox 96® Non-Radioactive Cytotoxicity Assay (Promega, Madison, WI, USA).

### Cell lines

CLDN18.1 CHO, CLDN18.2 CHO, panc02, CLDN18.2 panc02, HEK293T and Palt-E cells were purchased from the Cell Bank of the Shanghai Institute of Biochemistry and Cell Biology. CLDN18.1 CHO, CLDN18.2 CHO, panc02 and CLDN18.2 panc02 cells maintained at 37 °C with 5% CO_2_ in RPMI 1640 medium (Gibco) supplemented with 10% fetal bovine serum (FBS, Gibco), 100 U/mL penicillin and 100 µg/mL streptomycin (HyClone), and HEK293T and Plat-E cells were cultured in DMEM (Gibco) supplemented with 10% FBS and antibiotics.

### Lentivirus production and CLDN18.2 panc02 cell construction

The full-length human claudin 18.2 (accession number NM_001002026.3) gene was cloned and inserted into a lentiviral vector containing an internal ribosomal entry site (IRES) and a puromycin selectable marker. To produce the lentiviral supernatant, 293T cells were cotransfected with lentiviral vectors, the PSPAX plasmid containing the sequence for HIV-1 gag-pol, and the VSV-G plasmid containing the sequence for the vesicular stomatitis virus glycoprotein envelope using polyethyleneimine (PEI) transfection reagent (Sigma, USA) according to the manufacturer’s instructions. The supernatant containing the lentivirus was collected 48 and 72 h later. For transduction, 0.5 × 10^6^/mL PANC02 cells were plated in complete media in 6-well plates. After the addition of viral supernatant with polybrene (8 μg/ml), the cells were spun and incubated at 37 °C in 5% CO_2_. Puromycin (1 μg/ml) was added 72 h later, and CLDN18.2 expression was detected 48 h later. CLDN18.2 PANC02 cells were maintained in complete media supplemented with 0.5 μg/ml puromycin.

### Transduction and expansion of human T cells

Peripheral blood mononuclear cells were isolated with Ficoll (Solarbio, Beijing, China), and T lymphocytes were isolated with an EasySep™ Human T-Cell Isolation Kit (STEMCELL) and activated with 25 µL/mL human CD3/CD28/CD2 T-cellcell activator (STEMCELL). Forty-eight hours later, T lymphocytes were transduced with lentivirus in 24-well plates precoated with retronectin. On day 4, the transduced T cells were maintained in complete media (X-vivo medium (Lonza, Switzerland), 100 units/mL penicillin and 100 μg/mL streptomycin) supplemented with IL-7 (10 ng/mL; PeproTech), IL-15 (5 ng/mL; PeproTech), or IL-21 (30 ng/mL; PeproTech), and the medium was changed every 2–3 days. CAR-T cells were collected for subsequent experiments at 10–14 days post transduction.

### Retrovirus production and transduction of murine T cells

Retroviral supernatant was produced by Plat-E cells with the GeneJuice® Transfection Reagent (Merck). Similarly, 48- and 72-h supernatants were collected for subsequent transduction. Murine T lymphocytes were isolated with a MojoSort™ Mouse CD3 T-Cell Isolation Kit (BioLegend) from splenocytes obtained from C57BL/6J mice and stimulated with Dynabeads Mouse T-Activator CD3/CD28 (Thermo Fisher) for 48 h. Activated murine T cells were transduced via the same protocol used to transduce human T cells. Murine T lymphocytes were subsequently expanded in complete medium (RPMI-1640 (Gibco), 10% FBS (Gibco), 2 mM GlutaMAX (Gibco), 100 μM β-mercaptoethanol (Gibco), 1% penicillin and streptomycin, and IL-2 (30 units/mL; PeproTech)), and the medium was changed every 2 days. On day 5, expanded T cells were collected and used for in vitro and in vivo assays.

### Cell viability assay

The viability of the mouse pancreatic cancer cell line CLDN18.2 was detected with a Cell Counting Kit (CCK-8; Shanghai, Yeasen). CLDN18.2 PANC02 cells were collected and plated in 96-well plates at 2 × 10^3^ cells/well overnight, after which the cells were subjected to various doses of irradiation. After 48 h, the cell viability assay was performed according to the manufacturer’s instructions. Thereafter, the optical density of each well was recorded at 450 nm via a microplate reader.

### Quantitative real-time PCR

RNA was extracted from cells and tissues via RNeasy Micro Kits (Qiagen, Germany) and reverse-transcribed into cDNA via TransScript All-in-One First-Strand cDNA Synthesis SuperMix for qPCR (TransGen, Beijing, China). Quantitative real-time PCR was performed via PerfectStart® Green qPCR SuperMix (TransGen, Beijing, China) and a CFX96 Touch Real-Time PCR System (Bio-Rad, USA) according to the manufacturer’s instructions. The sense and antisense primers for the following genes were purchased from Sangon (Shanghai, China): mGAPDH, 5′-CATCACTGCCACCCAGAAGACTG-3′, and 5′- ATGCCAGTGAGCTTCCCGTTCAG-3′; mBAX, 5′-AGGATGCGTCCACCAAGAAGCT-3′, and 5′-TCCGTGTCCACGTCAGCAATCA-3′; mCDKN1A, 5′- TCGCTGTCTTGCACTCTGGTGT-3′, and 5′-CCAATCTGCGCTTGGAGTGATAG-3′; mAURKA, 5′-TCATCCTGGCTCTGAAGGTGCT-3′, and 5′-CCATACAGCCTGAGGATGTTGG-3′; mAURKB, 5′-CTTCTACGACCAGCAGAGGATC-3′, and 5′-GGCATCTGACAGTTCCTCCATG-3′; mCXCL9, 5′- CCTAGTGATAAGGAATGCACGATG-3′, and 5′- CTAGGCAGGTTTGATCTCCGTTC-3′; mCCL2, 5′-GCTACAAGAGGATCACCAGCAG-3′, and 5′GTCTGGACCCATTCCTTCTTGG-3′; hCLDN18.2, 5′-GACTGCCTGTCAGGGCTTG-3′, and 5′-GCTCTCTCGGACACAGGAG-3′.

### Cytotoxicity assay

The cytotoxicity of the CAR-T cells was determined via a CytoTox 96® Non-Radioactive Cytotoxicity Assay (Promega, USA). The tumor cells served as target cells and were plated in 96-well plates at 2 × 10^3^ cells/well overnight. The effector cells were cocultured at the indicated effector/target (E:T) ratios in triplicate on plates. After 18 h, the substrate mixture was added to each well and incubated at room temperature for 30 min in the dark. The absorbance was measured at 490 nm using a microplate reader.

### Mice

NSG mice (male, 6–8 weeks) and C57BL/6 mice (female, 6–8 weeks), weighing 16–20 g, were purchased from GemPharmatech (Nanjing, China) and housed at the Animal Center of Shenzhen University Medical School (Shenzhen, China).

### Animal experiments

To investigate the antitumor effect of radiotherapy combined with CAR-T cells, 1 × 10^6^ CLDN18.2 PANC02 cells were subcutaneously inoculated into 6–8-week-old male NSG mice. Seven days later, the mice were randomly and blinded assigned to four treatment groups (*n* = 4), namely, the control, radiotherapy, CLDN18.2 CAR-T and synergy groups. The radiotherapy and synergy groups were locally exposed to a single dose of 4 Gy, with a lead shield protecting the rest of the mice. Control T cells or CAR-T cells (1 × 10^6^ in 200 µL) were injected intravenously at 7 days after radiation treatment. The body weights and tumor volumes of the mice were recorded every other day. The tumor size was measured via a digital caliper and calculated via the formula π ÷ 6 × H × W × L. Peripheral blood was collected at the indicated time points to measure the expansion of infused T cells (CD3 + CD8 + NGFR + ) and effector T cells (CD3 + NGFR + CD45RA + CCR7-) via flow cytometry.

To establish a bilateral tumor model, 2 × 10^6^ CLDN18.2 PANC02 cells were subcutaneously injected into the right (local tumors) and left (distant tumors) flanks of C57BL/6 mice on day 0. On day 7, the mice were randomized into four treatment groups: the control (*n* = 5), radiotherapy (*n* = 6), CLDN18.2 CAR-T (*n* = 6) and synergy (RT + CAR-T, *n* = 8) groups. The right-sided tumors of the two groups (radiotherapy and synergy groups) were locally irradiated with a single dose of 4 Gy. On day 8, local tumors were administered control T cells or CLDN18.2 CAR-T cells (1 × 10^6^ in 50 µL) by intratumoral injection, and tumor growth and body weight were monitored every other day. One week after T-cell treatment, 3 mice in each group were randomly selected and sacrificed. Tumors (local and distant) were collected and processed for transcriptome analysis. All the mice were euthanized at 28 days post-T-cell infusion, and the tumor tissue was collected to measure the infiltration of infused T cells (mCD3 + mCD8+NGFR + ) and endogenous T cells (mCD3 + mCD8+NGFR-) via immunohistochemistry.

### Flow cytometry analysis

Single-cell suspensions of cells were stained with antibodies at room temperature for 15 min or at 4 °C for 30 min in the dark. The following antibodies used for flow cytometry analysis were obtained from Biolegend: APC/Cyanine7 anti-human CD3 (Clone HIT3a), PerCP/Cyanine5.5 anti-human CD4 (Clone RPA-T4), PE/Cyanine7 anti-human CD8 (RPA-T8), Brilliant Violet 605™ anti-human CD45RA (Clone HI100), FITC anti-human CD197 (CCR7) (Clone G043H7), APC anti-human EGFR (Clone AY13), APC/Fire™ 750 anti-mouse CD3 (Clone 17A2), Brilliant Violet 510™ anti-mouse CD8 (Clone 53--6.7), APC anti-mouse CD4 (Clone GK1.5), and PE/Cyanine7 anti-human CD271 (NGFR) (Clone ME20.4) antibodies. Flow cytometry data were collected on a 10-color CytoFLEX cytometer (Beckman Coulter, USA).

### IHC

The tumor sections were fixed in 4% formalin for 10 min, air dried, pretreated with 0.3–3% H_2_O_2_ for 10 min, and blocked for 1 h at room temperature. After blocking, 400 μl of primary antibody was applied overnight at 4 °C. The primary antibodies used included the following: CCL2/MCP-1 rabbit pAb (ABclonal, 1:100) and anti-CXCL9 antibody (Abcam, 1:100). CD8α (D4W2Z) XP® rabbit mAb (CST, 1:200) and anti-p75 NGF receptor antibody (Abcam, 1:50) were used. Biotinylated secondary antibodies were purchased from Abcam. The membranes were then incubated with secondary antibodies for 30 min at room temperature. The sections were stained with 3,3′-diaminobenzidine and hematoxylin for 1–10 min, washed 3 times in water, dehydrated twice in 95% ethanol for 10 s, incubated in 100% ethanol twice, and incubated in xylol three times before mounting the sections with coverslips with mounting medium (Sigma). ImageJ was used to quantify immunobiological staining.

### RNA sequencing and transcriptome analysis

One week and four weeks after CAR-T-cell therapy, local tumors and abscopal tumors from the mice were excised and quickly frozen in liquid nitrogen. Total tumor RNA was extracted via RNeasy Micro Kits (Qiagen, Germany) following the manufacturer’s instructions and then used for RNA-seq (Novogene, Beijing, China). Differential expression analysis was performed with the DESeq2 Bioconductor package. Gene Ontology (GO) and KEGG enrichment analyses were performed via the “Gene Ontology Resource” and “KEGG” databases, respectively. Gene set enrichment analysis (GSEA) was performed via GSEA software. Genes with a > 1 log2fold change and an FDR < 0.05 were considered significantly different between the CAR-T and synergy groups. The DEGs involved in leukocyte migration pathways identified via RNA-seq were independently confirmed via qRT‒PCR.

### Statistical analysis

All the data are presented as the means ± Standard Deviations (SDs), and at least three independent experiments performed in duplicate. The data were analyzed via GraphPad Prism 7. The statistical significance of differences between the two groups was analyzed by two-tailed Student’s *t* test, and multiple comparisons were evaluated by two-way ANOVA. *p* < 0.05 was considered statistically significant (ns *p* > 0.05, *p < 0.05, **p < 0.01, ***p < 0.001).

## Supplementary information

supplementary figures

## Data Availability

Data are available upon reasonable request.
