# Supplementary material for: Systemic tumor regression with synergy therapy: radiotherapy and CAR-T
Source: Cell Death Discov. 2024 Nov 22;10:479. doi: 10.1038/s41420-024-02245-3 (PMC11584735; doi:10.1038/s41420-024-02245-3)
Supplement: Supplementary file 1 — supplementary figures [file 41420_2024_2245_MOESM1_ESM.docx]

**Supplementary Materials for**

**Systemic tumor regression with synergy therapy: radiotherapy and CAR-T**

Xingyu Ma^1^, Wei Zhang^1^, Miao Zeng^1^, Teeranut Asavasupreechar^1^, Kang Synat^1^, Yisheng Li^2,3^* & Li Yu^1,2^*

***Correspondence**: [ysli@haoshibio.com](mailto:ysli@haoshibio.com); [yuli@szu.edu.cn](mailto:yuli@szu.edu.cn). Tel: 0755-21839215.

^1^ Department of Hematology and Oncology, Shenzhen University General Hospital, International Cancer Center, Hematology Institution of Shenzhen University, Shenzhen University Health Science Center, Shenzhen Clinical Research Center for hematologic disease, Shenzhen University, Xueyuan AVE 1098, Shenzhen 518000, China.

^2^ Biomedical Laboratory, Shenzhen University-Haoshi Cell Therapy Institute, Shenzhen, China.

^3^ R&D Department, Shenzhen Haoshi Biotechnology Co., Ltd, Shenzhen, China.

**This file includes: Figures S1 to S6.**


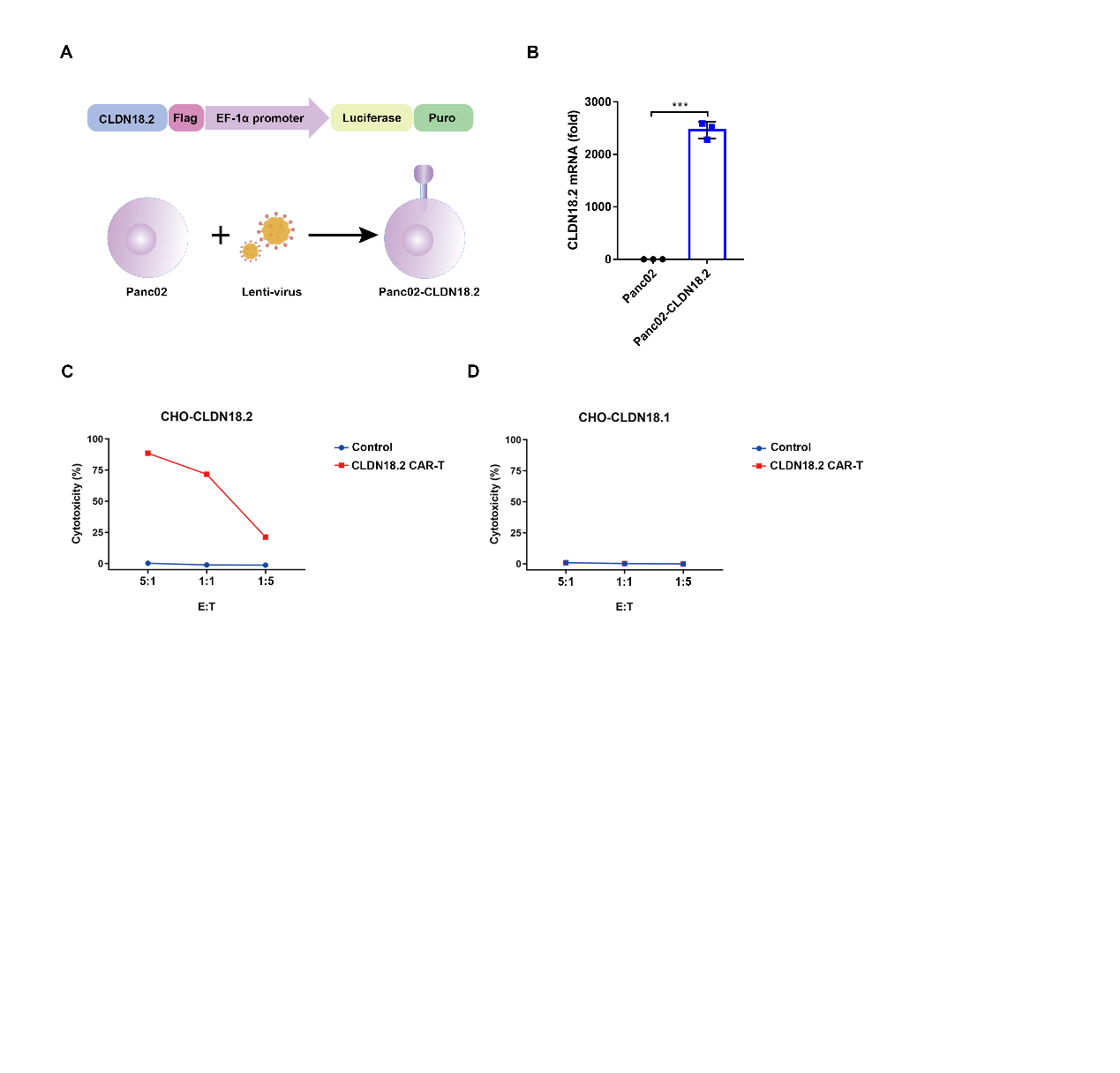


**Figure S1: CLDN18.2 CAR T cells lyse pancreatic cancer cells in a CLDN18.2-dependent manner.** (A) Schematic of the vectors encoding CLDN18.2 and sketch of CLDN18.2 panc02 construction, (B) and CLDN18.2 mRNA was analyzed by q-PCR. (C) CLDN18.2 CHO or (D) CLDN18.1 CHO cells incubated with CLDN18.2 CAR or control T cells at various E:T ratios for 18 hr, followed cytotoxicity was quantified. Data in B presented as the mean ± SD, n = 3, statistically signiﬁcant differences were calculated by two-tailed Student’s t-test. ***p<0.001.


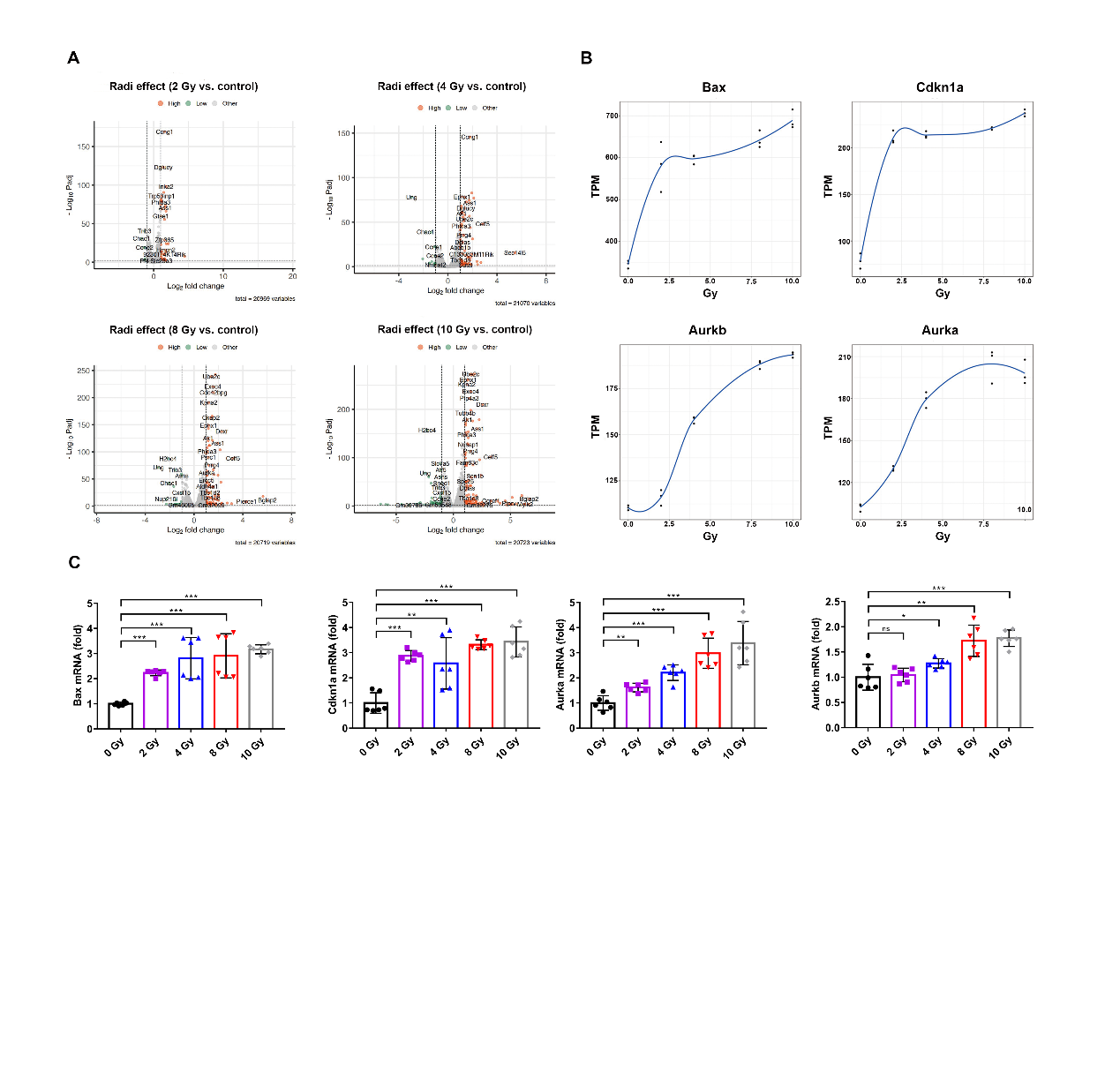


**Figure S2:** **RT induced Pancreatic Cancer cells apoptosis without dose-independent.** (A) Volcano plots displaying names of the genes that were significantly (FDR=0.05) and ≥2-fold downregulated (left) or upregulated (right) in cells 6 hr after 2, 4, 8, 10 Gy RT exposure. (B) Correlation plots of expressions of BAX, CDKN1A, AURKA and AURKB genes. (C) Analysis of Indicated genes mRNA expression by q-PCR. Data in C presented as the mean ± SD, n = 3, statistically signiﬁcant differences were calculated by two-tailed Student’s t-test. *p<0.05, **p<0.01, ***p<0.001.


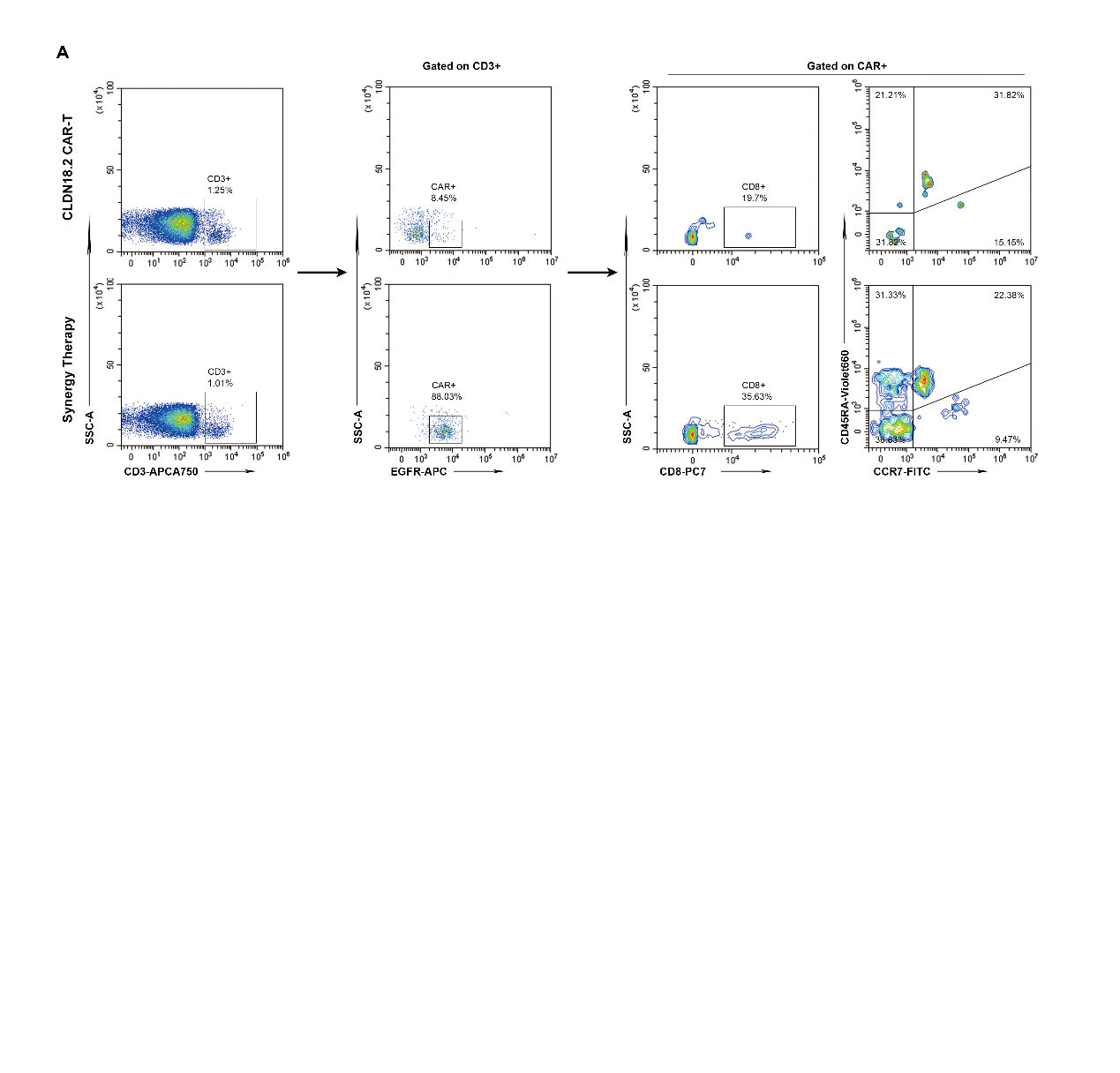


**Figure S3: Phenotypic analysis of CAR-T cells.** (A) Representative flow cytometry plots showing the expression of CD8+ and effector T cells in CLDN18.2 CAR-T group and synergy therapy group at day 7 post CAR-T injection (n = 4).


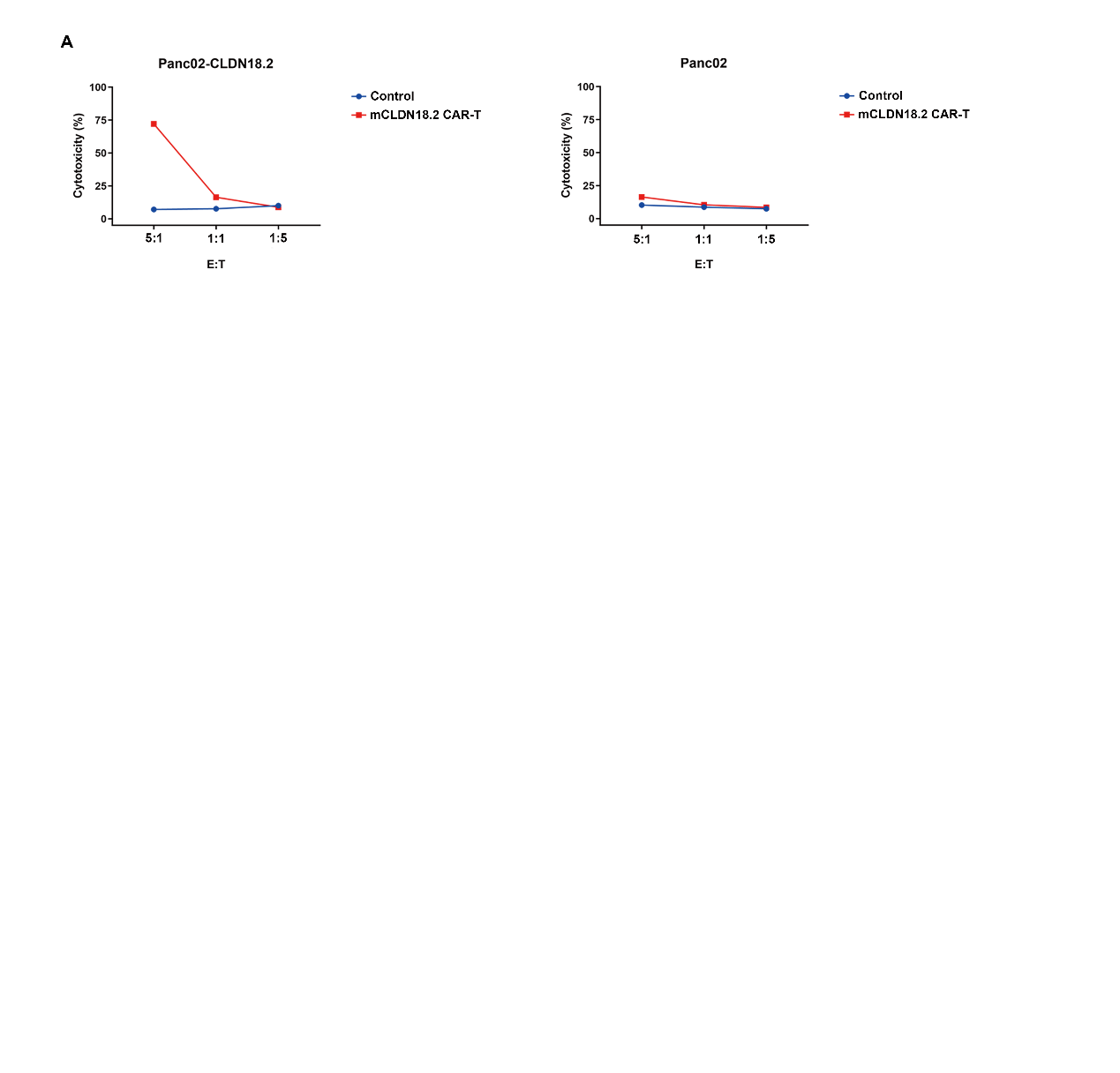


**Figure S4: mCLDN18.2** **Speciﬁc CAR-T cells are active against pancreatic tumor cells *in vitro*.** (A) mCLDN18.2 CAR-T or control T cells were incubated with CLDN18.2 panc02 or panc02 cells at indicated E:T ratios for 18 hr, followed cytotoxicity was quantified.

**
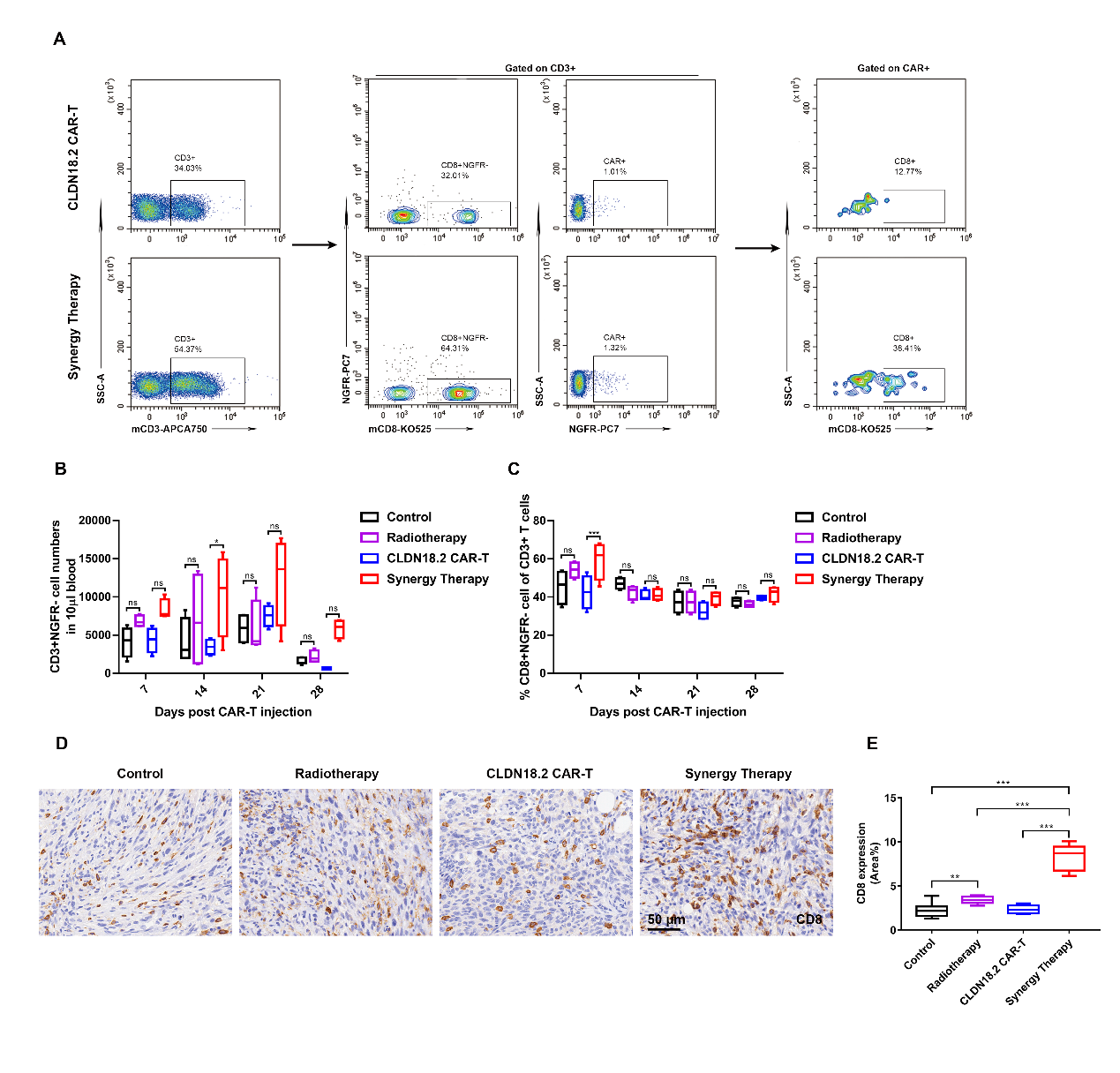
**

**Figure S5: RT and CAR-T synergy therapy enhanced infiltration of CAR-T.** (A) Representative flow cytometry plots showing the expression of CD8+ T cells in CLDN18.2 CAR-T group and synergy therapy group at day 7 post CAR-T infusion (n = 4). (B) Numbers of CD3+NGFR- cell in murine peripheral blood was drawn at indicated after CAR-T cell infusion. Data indicate the mean number of T cells per 10 μl of blood as measured by ﬂow cytometry. (C) Proportion of CD8+NGFR- in murine peripheral blood indicated days post CAR-T injection. (D) Immunohistochemistry of local tumor for CD8. (E) Quantification of CD8 expression (area%) by Image J. Statistically signiﬁcant differences were calculated by two-tailed Student’s t-test. Data in B, C and E presented as the mean ± SD, n = 4. *p<0.05, **p<0.01, ***p<0.001.

**
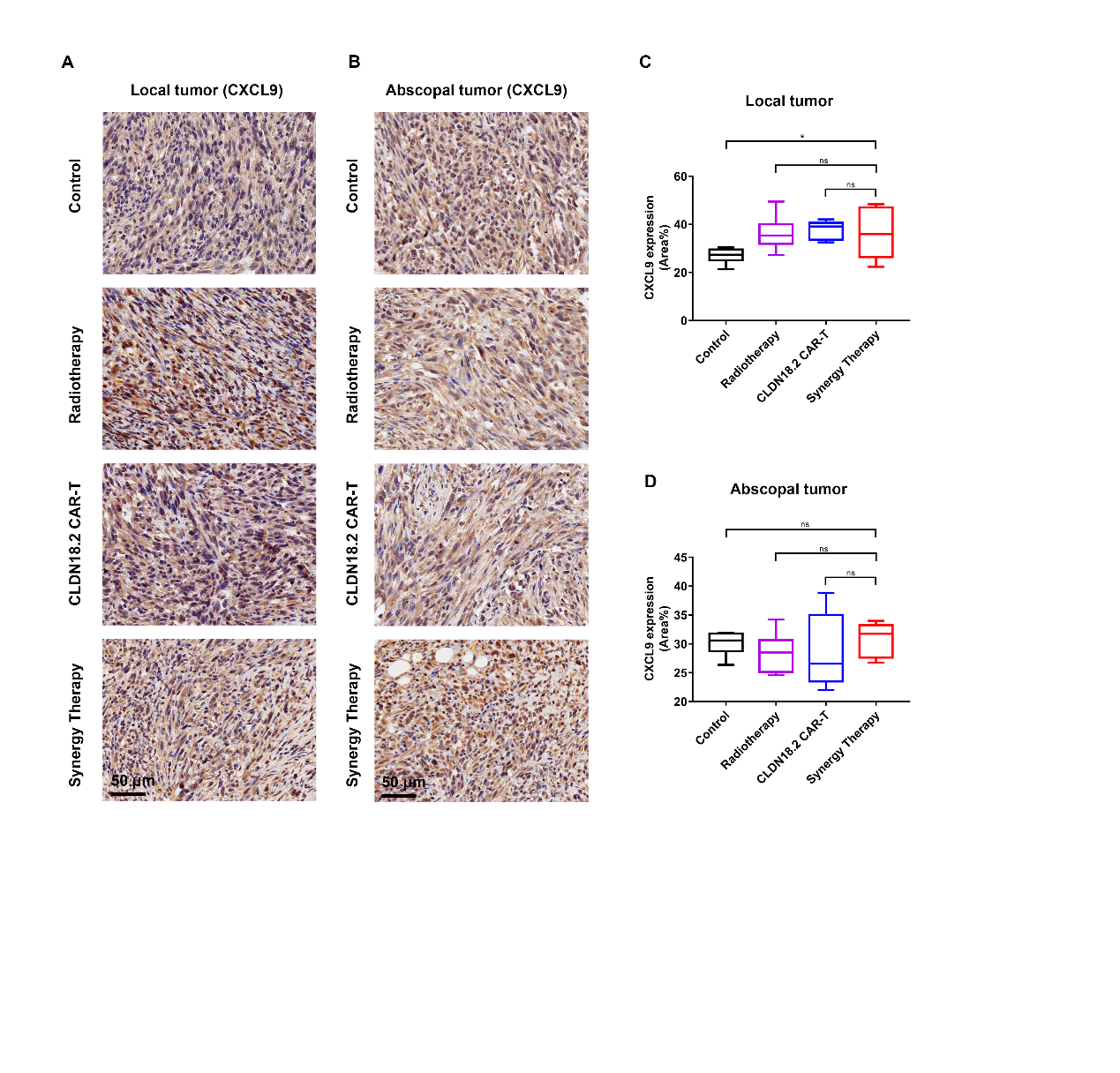
**

**Figure S6: Synergy therapy had little effect on CXCL9 expression.** Immunohistochemistry of local tumor (A) and abscopal tumor (B) for CXCL9. Quantification of CXCL9 expression (area%) local tumor (C) and abscopal tumor (D) by Image J. Statistically signiﬁcant differences were calculated by two-tailed Student t test. Data in C and D presented as the mean ± standard deviation. *p<0.05.
